# Acute appendicitis secondary to hypertriglyceridemia‐induced acute pancreatitis: A case report

**DOI:** 10.1002/ccr3.4798

**Published:** 2021-09-15

**Authors:** Dhruba Kadel, Sabin Chaulagain, Bikash Raj Thapa, Angela Basnet, Shashinda Bhuju

**Affiliations:** ^1^ Department of General Surgery Scheer Memorial Adventist Hospital Banepa Nepal; ^2^ Department of Internal Medicine Scheer Memorial Adventist Hospital Banepa Nepal; ^3^ Department of Radiology, Consultant Radiologist National Academy of Medical Sciences National Trauma Center Kathandu Nepal

**Keywords:** acute appendicitis, acute pancreatitis, hypertriglyceridemia

## Abstract

Hypertriglyceridemia led acute pancreatitis secreted exudative fluid tacked to the right iliac fossa may cause irritation of retroperitoneum leading to acute periappendicular inflammation and acute appendicitis.

## Graphical abstract

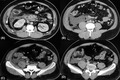

## 1 INTRODUCTION

The colonic involvement in acute pancreatitis is quite rare and most commonly occurs in the adjacent colonic part including transverse and splenic flexure colon. This case showed that extrinsic, secondary acute appendicitis could be as a complication of acute pancreatitis.

Acute pancreatitis is one of the most common diagnoses in the emergency room for acute abdominal pain.^1^ Many causative agents have been recognized in development of acute pancreatitis including gallstone, alcohol, endoscopic retrograde cholangiopancreatography, some metabolic conditions, infection, and hypertriglyceridemia.^2^ Even though there is no consensus on diagnostic threshold of triglyceride level, nonfasting triglyceride levels greater than 177mg/dl (2mmol/l) are considered a risk factor of acute pancreatitis.^3^

Acute appendicitis is a leading surgical reason for patients to visit emergency department.^1^ Acute appendicitis is typically caused by direct luminal obstruction or infection and may be influenced by genetic or environmental factors, but largely remains unknown.^4^

In this brief case report, we present a 39‐year‐old patient who presented with acute pancreatitis due to hypertriglyceridemia who concurrently developed acute appendicitis.

## 2 CASE REPORT

A 39‐year‐old gentleman presented to emergency department with 5 h of diffuse abdominal pain, localized to epigastric and periumbilical regions, associated with nausea and one episode of vomiting. He endorsed eating a diet of saturated fats from meat, greasy, and oily foods. His physical examination was significant for epigastric and periumbilical tenderness without rebound, guarding, or rigidity. The rectal examination was notable for the absence of blood and an evacuated rectal vault.

**FIGURE 1:**
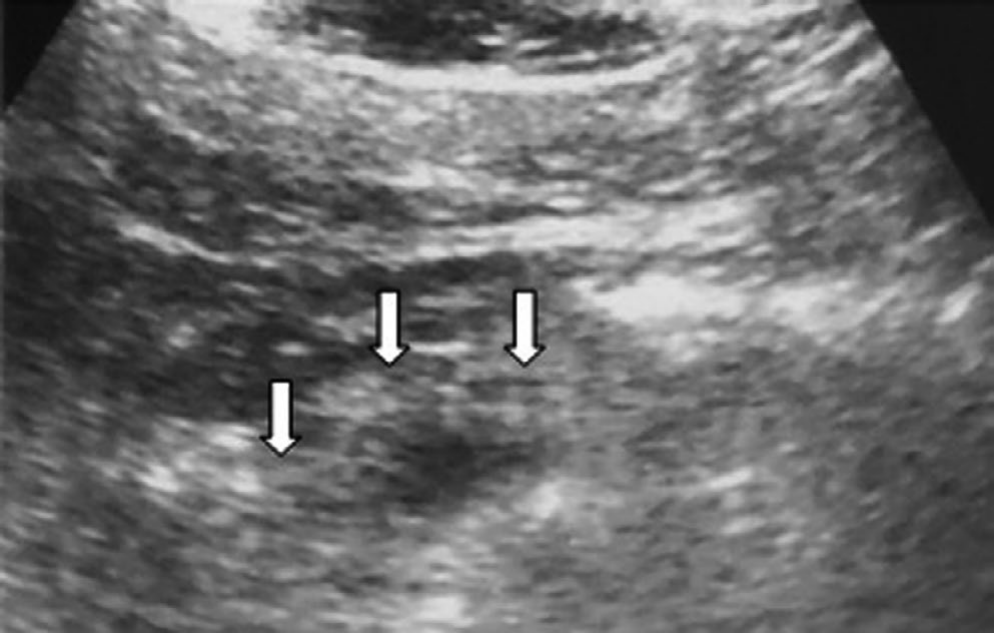
Acute pancreatitis. USG upper abdomen demonstrates heteroechoic echotexture of pancreas (arrows showing head; neck and body of pancreas)

Objective parameters were as follows: Total count: 16,300/cumm^3^ with neutrophils: 70%, hematocrit: 43%, platelet count: 330,000/cumm^3^, Serum lipase: 2100 U/L (Vitros lipase; normal range 23–300 U/L), lactate dehydrogenase: 225 U/L, calcium: 7.5 mg/dl, Blood Urea Nitrogen: 12.1 mg/dl, Random blood sugar: 120 mg/dl triglycerides: 523 mg/dl. The liver function test, renal function test, and coagulase test were within normal limit. Ultrasonography (USG) of abdomen showed edematous with heterogeneous echotexture of pancreas, without other significant abnormality (Figure 1). A postero‐anterior chest X‐ray found consolidation in the right lower zone with a minimal pleural effusion. Hence, patient was admitted to the hospital with the diagnosis of hypertriglyceridemia‐induced acute pancreatitis (Ranson's score = 1) with right lower zone pneumonia and pleural effusion. Conservative management with empirical antibiotic Piperacillin/tazobactam was commenced.

**FIGURE 2:**
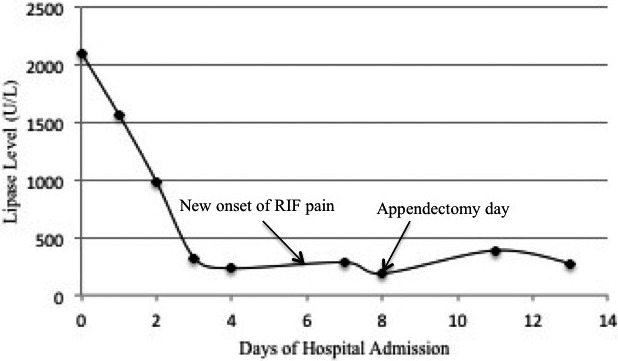
Serum lipase level. *X* axis: Days of hospital admission; *Y* axis: Serum lipase level with reagent vitros lipase

**FIGURE 3:**
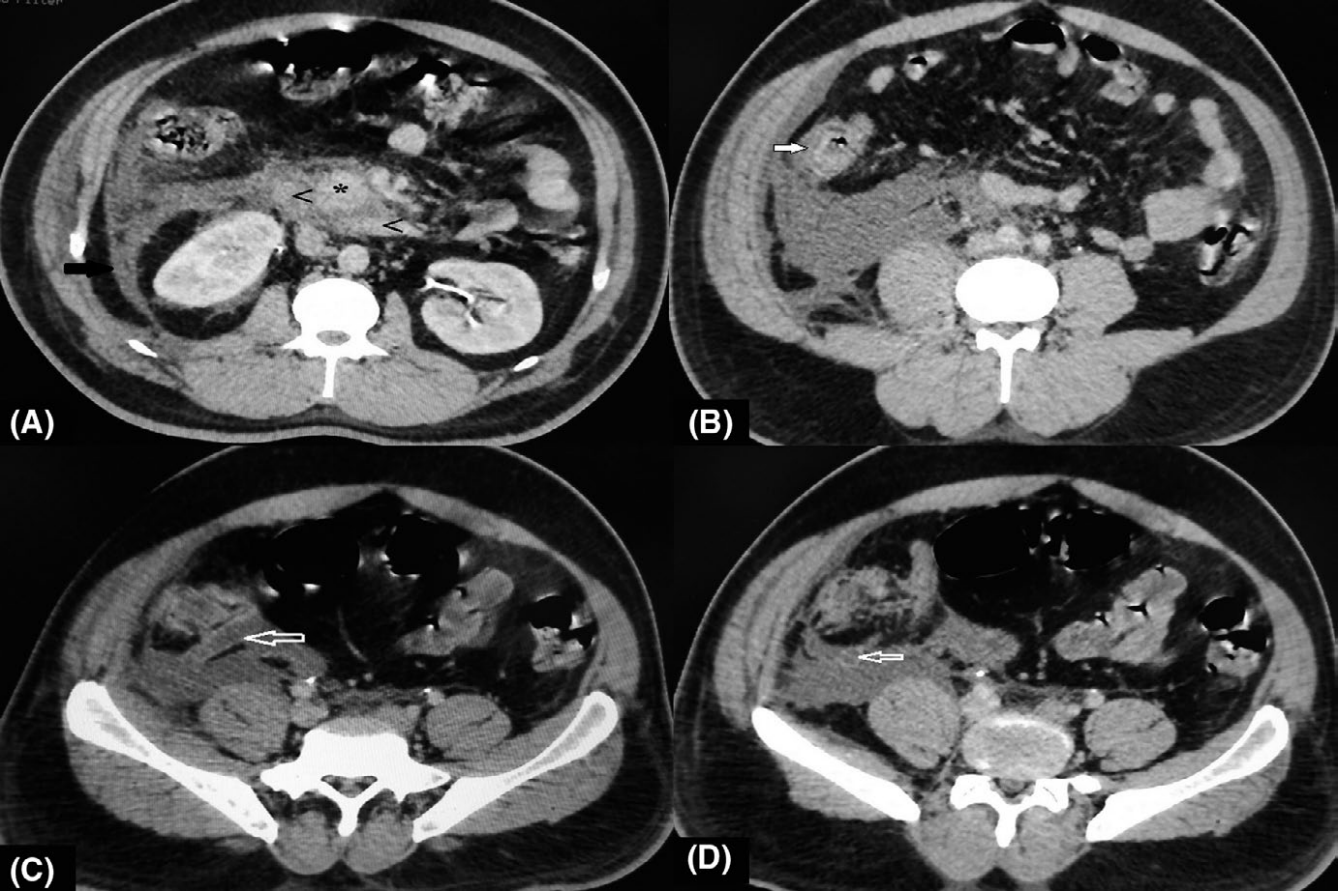
Acute pancreatitis and acute appendicitis. Contrast CT of abdomen: (A) demonstrates strandings along the uncinate process of pancreas (*) along with fluid collection in peripancreatic and periduodoneal (>> second and third part of duodeneum) region of retroperitoneum. Fluid in pararenal retroperitoneum in right side limited posteriorly by lateralconal fascia (black arrow). (B) Mural stratification of caecum with symmetrical regular circumferental edematous wall. Fluid collection in retroperitoneum is displacing caecum anteriorly. (C, D) demonstrates retrocaecal appendix is distended (11 mm) with fluid and with mild enhancing smooth wall and with periappendiceal fluid collection

Over the following 48 h, the patient's Ranson's score deteriorated to three, serum calcium decreased and partial pressure of oxygen on arterial blood gas analysis fell. Despite the worsening in these parameters, the serum lipase decreased (Figure 2). An abdominal contrast‐enhanced computed tomography (CECT) demonstrated the features suggestive of acute pancreatitis (Modified CT severity index; CTSI 6) with acute appendicitis (Figure 3a–d). However, medical and surgical team opted for a nonsurgical intervention, where Piperacillin/tazobactam was switched to meropenem and a somatostatin analogue (Inj. Octreotide 200 mg SC Q8h) was added for supportive therapy.

**FIGURE 4:**
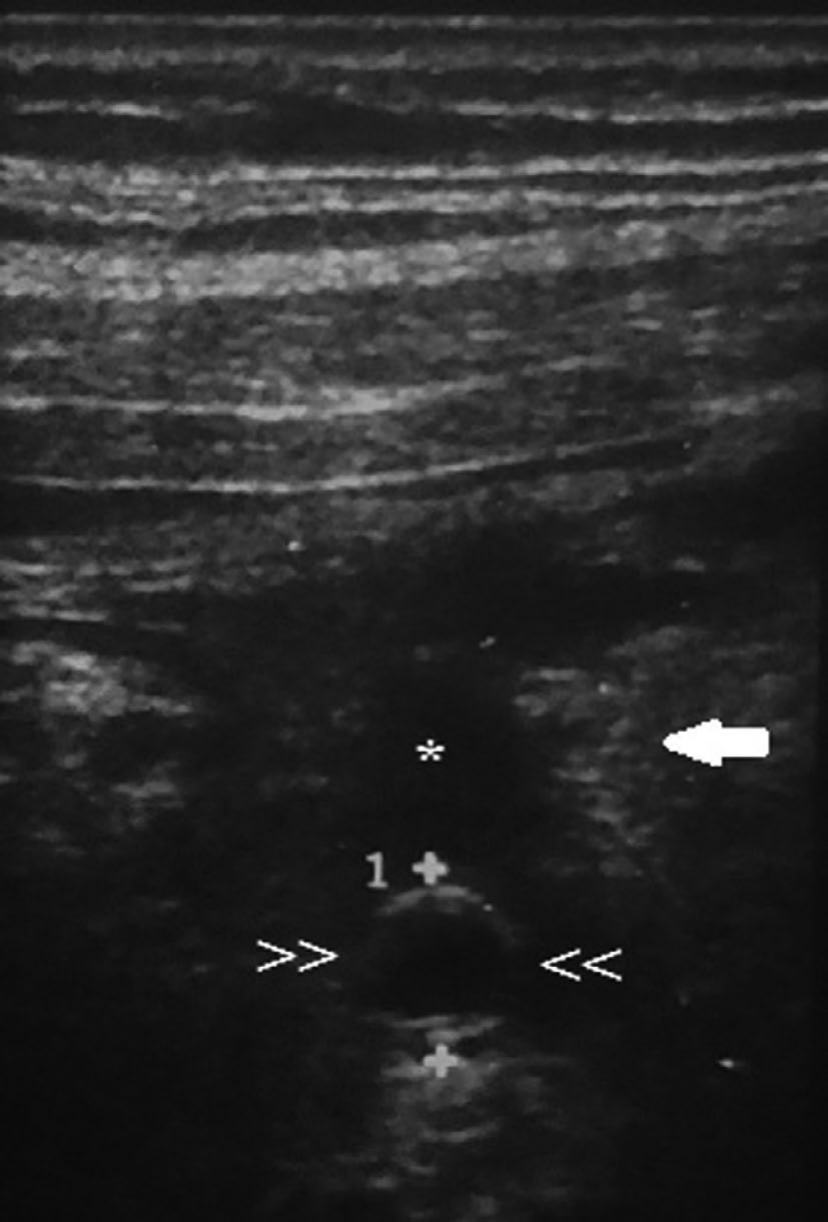
Acute appendicitis. USG of right iliac fossa region shows: target appearance of fluid filled appendix (>> <<) with periappendiceal free fluid (*) collection and omental thickening (white arrow)

After the initial improvement of the patient's clinical status his abdominal pain worsened and localized to the right iliac fossa. Upon repeat physical examination and ultrasound (Figure 4) did not show improvement of acute appendicitis. Hence, an open appendectomy with abdominal drain placement was performed on the ninth day of admission.

**FIGURE 5:**
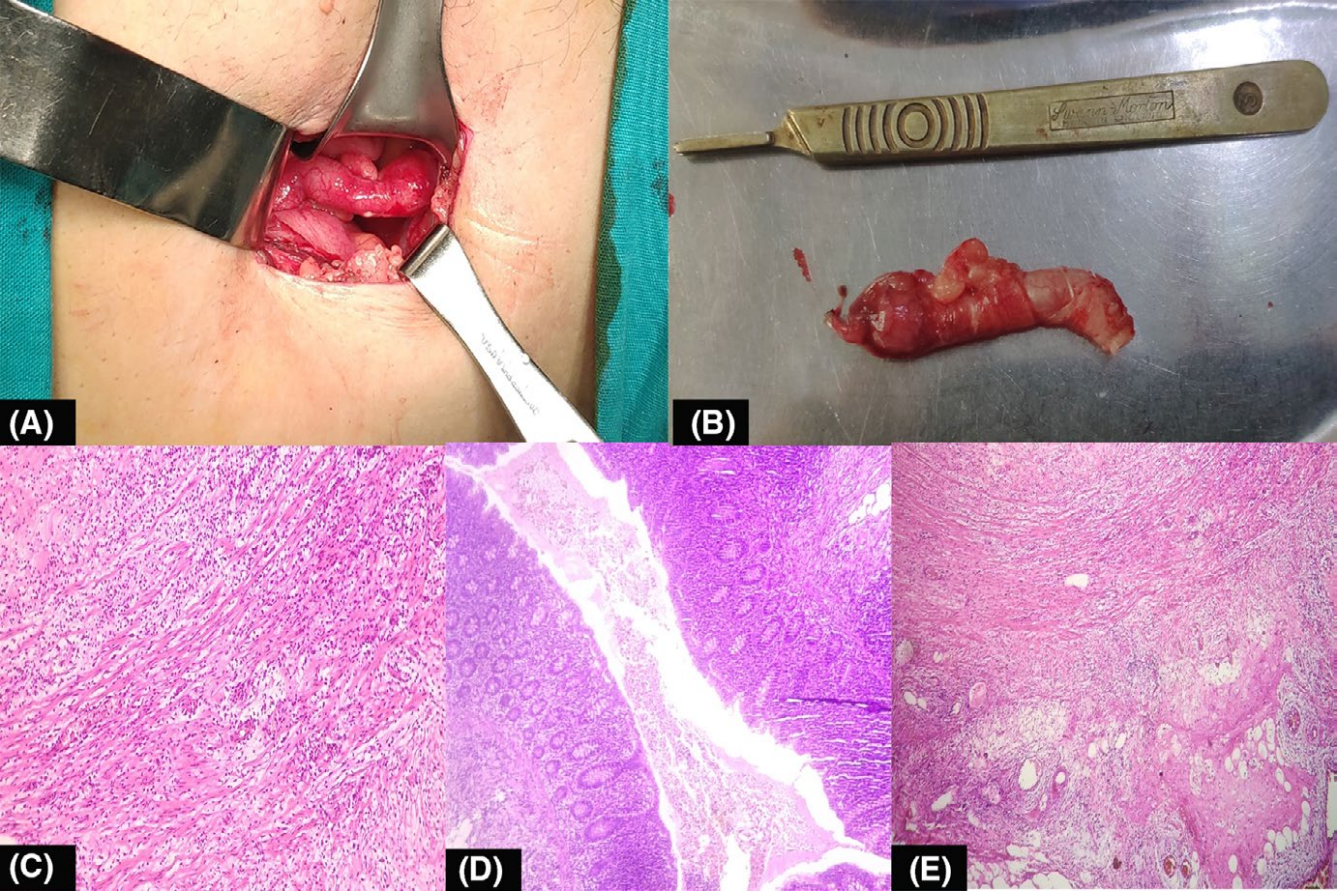
Macroscopic and microscopic features of acute appendicitis with periappendiceal acute inflammation: (A) Intraoperative image of inflamed Vermiform Appendix. (B) Inflamed Vermiform Appendix measures 7 cm × 1.5 cm. (C) Muscularis propria layer showing moderate to dense neutrophilic infiltration H & E stain (D) Spared lumen of Appendix showing no neutrophilic infiltration H & E stain (E) Neutrophilic infiltration seen in mesoappendix H & E stain

The operative findings were notable for an inflamed, retrocecal appendix. The base of appendix was non‐inflamed. Approximately 100 ml of peritoneal fluid was aspirated from the periappendicular space. (Figure 5a, b). The peritoneal fluid was measured for lipase and amylase, both were normal. Additionally, the bacterial culture of the fluid was sterile at 5 days. The drain output was mixture of serosanguineous and ascitic in nature, which gradually decreased and removed on postoperative day three. This subsequent recovery was unremarkable. The histopathology analysis of Vermiform Appendix demonstrated neutrophilic infiltration of the muscularis propria layer and mesoappendix. There was sparing of the mucosa and lumen. These findings were consistent with acute appendicitis with periappendicular acute inflammation. (Figure 5c–e). The patient followed up in the postoperative clinic on day 14 and was found to be symptom‐free.

## 3 DISCUSSION

In summary, this 39‐year‐old man was diagnosed with hypertriglyceridemia‐induced acute pancreatitis after an otherwise negative review of personal, social, and family history risk factors and biochemical workup. Hypertriglyceridemia is a known risk factor for acute pancreatitis.^3^

As two distinct pathological processes were concurrently observed in this patient, the question arises was this due to two separate processes or one as the complication of other? One must be cautious as confirmation bias may be utilized following the recovery of the patient with a surgical intervention. While exploring the published literatures, only handful of cases of periappendicitis and appendicitis in different patients as the complication of acute pancreatitis have been reported.^5^, ^6^

One should be aware that there are cases in the literature of similar presentations where amylase and lipase are elevated in the clinical context of acute appendicitis.^7^, ^8^ Based on the biochemical profile of a serum lipase elevated more than three times upper limit of normal, in the context of abdominal pain, one can safely conclude that the patient had acute pancreatitis. This diagnosis is supported and validated by the intravenous contrast CT findings when performed on specific time.^9^

From a radiological perspective, ultrasound has a sensitivity of 86% and a specificity of 81%, compared to computed tomography's sensitivity of 94% and specificity of 95%, for diagnosing acute appendicitis.^10^ Ultrasound performed on this patient at the time of admission showed bulky and heterogeneous echotexture of pancreas but not the features of acute appendicitis. However, both CT and repeat ultrasound were suggestive of acute appendicitis and this fact was confirmed by the histopathological analysis.

We believe the clinical phenomenon observed in this case could be because of the following:

1. Hypertriglyceridemia led to acute pancreatitis
2. Peri‐pancreatic fluid and retroperitoneal fluid accumulated, as was observed on the CT scan was exudative fluid of pancreas
3. Retroperitoneal fluid tracked to the right iliac fossa caused irritation of retroperitoneum leading to periappendicular inflammation
4. Acute periappendicular inflammation gradually progressed to acute appendicitis.
5. Per operative observed intraperitoneal fluid may be only reactive fluid of acute appendicitis but not the pancreatic exudate, thus normal amylase and lipase was seen upon analysis

Once again, the patient may have developed appendicitis incidentally and unrelated to the acute pancreatitis. However, given the time course, it makes sense that there is a unifying pathophysiological mechanism.

From a management perspective, we do not believe that there was an antibiotic failure in this case. Frankly, given the aforementioned mechanism, antibiotics may not have even been required in the management of this patient's appendicitis. Whether or not somatostatin helped this patient is unknown. We added this therapy based on other case reports.^6^

In summary, this 39‐year‐old patient with hypertriglyceridemia‐induced acute pancreatitis concurrently developed acute appendicitis. We think there is a unifying mechanism that we have attempted to hypothesize. The exudative fluid secreted by pancreas in retroperitoneal space, which was tracked to RIF could be the precipitating factor to induce periappendicitis and appendicitis. However, further studies should focus on further elucidation of a pathophysiological mechanism.

## CONFLICT OF INTEREST

The authors declare there is no conflict of interest.

## AUTHOR CONTRIBUTIONS

Dr. Dhruba Kadel involved in study concept design and writing paper. Dr. Sabin Chaulagain and Dr. Bikash Raj Thapa involved in exploring the information regarding acute pancreatitis and acute appendicitis, radiological and histopathological interpretation on patient's finding and helping to write paper. Dr. Angela Basnet and Dr. Shashinda Bhuju involved in collecting patient's information, follow‐up of patient's condition, and helping to write paper.

## CONSENT

Informed consent was obtained from patient for the use of relevant information for publication in journal.

## Data Availability

Data sharing is not applicable to this article as no new data were created or analyzed in this study.
